# Advancing multi-regional research in Canada through collaboration.

**DOI:** 10.23889/ijpds.v7i3.1882

**Published:** 2022-08-25

**Authors:** Nicole Yada, Michael Schull, Kimberlyn McGrail

**Affiliations:** 1 ICES; 2 University of British Columbia

## Objectives

COVID-19 accentuated the importance of breaking down data siloes and aligning incentives for data access, collection, and use. Health Data Research Network Canada (HDRN Canada) is responding to this challenge, bringing together people and organizations to transform health data use in Canada.

## Approach

HDRN Canada’s foundation is its partnership of 20 pan-Canadian, provincial and territorial data organizations that together are enabling multi-regional research. This is being enriched with HDRN Canada’s development of the Canada Health Data Research Alliance (HDR Alliance). The HDR Alliance coordinates expansion of sources and types of data available while retaining organizational independence. A project-based pilot approach is underway with two large pan-Canadian, longitudinal, consented cohort studies being linked at HDRN Canada sites. In addition, a collaboration with a pan-Canadian COVID19 clinical trials network is ensuring that clinical data are collected in ways that enables linkage with population-based administrative data.

## Results

HDRN Canada has created a single data access portal for researchers with information on over 500 datasets and supported 72 research projects to date. Work on the HDR Alliance adds data from the Canadian Partnership for Tomorrow’s Health and the Canadian Longitudinal Survey on Aging. The former includes 350,000 individuals, and survey data (including related to COVID-19), physical measures and genomics. The latter includes 50,000 individuals with survey data and physical measures. Four multi-region clinical trials are being planned with the support of HDRN Canada. Even with aligned incentives, challenges navigating data governance and access processes remain. Collaborations are necessary to address these complexities and enable access to richer data in an efficient and timely matter.

## Conclusion and Relevance

Strong partnerships are critical to unlocking the potential of Canada’s data assets and expertise. The HDR Alliance provides a collaboration mechanism to increase the “findability”, accessibility and utility of data assets, while addressing complex issues in the data landscape. This increases research opportunities and the impact of population-based, linkable data.

